# Preliminary Descriptive Characterization Reveals Physicochemical Differentiation of Tissue Mucus in *Crassostrea gigas*

**DOI:** 10.3390/ma19101912

**Published:** 2026-05-07

**Authors:** Shiyu Cui, Xiutong Wang, Na Liu, Xixi Wang

**Affiliations:** 1 Shandong Key Laboratory of Marine Environmental Corrosion and Bio-Fouling, State Key Laboratory of Advanced Marine Materials, Institute of Oceanology, Chinese Academy of Sciences, Qingdao 266071, China; serenity233@163.com (S.C.); liunaa163@163.com (N.L.); wangxixi@qdio.ac.cn (X.W.); 2 University of Chinese Academy of Sciences, Beijing 100049, China

**Keywords:** *Crassostrea gigas*, marine mucus, material characterization, microstructure

## Abstract

**Highlights:**

- **Labial Palps Mucus:** High protein and acidic amino acids form a cohesive molecular network and tightly packed microstructure that may contribute to barrier-like properties.
- **Mantle Mucus:** Rich in polysaccharides with dense hydrogen-bonding, its uniformity shows features that may be consistent with potential lubrication-related functions.
- **Gill Mucus:** A balanced composition creates a porous 3D network with a high surface area, which may potentially facilitate the process of respiratory and feeding functions.
- **Material Science Perspective:** This research offers a preliminary descriptive characterization of marine biopolymers providing foundational insights into their physicochemical properties.
- **Biomimetic Design:** The potential correlations between chemical composition and macroscopic functionality provide preliminary guidance for exploring novel tissue-specific biomimetic materials.
- **Ecological Adaptation:** The findings inspire our understanding of how bivalves adapt to complex marine environments through the potential tuning of biochemical secretions for defense and feeding.

**Abstract:**

Marine biomucus, a complex biomolecular gel, plays a pivotal role in defense against biofouling, mitigation of environmental stress, and regulation of biomineralization. This study conducts a comparative analysis of the physicochemical properties of mucus secreted by three distinct tissues—labial palps, mantle, and gills—of the Pacific oyster (*Crassostrea gigas*), alongside their freeze-dried counterparts. By integrating amino acid profiling, scanning electron microscopy (SEM), and Fourier-transform infrared spectroscopy (FTIR), we explored potential correlations between chemical composition, microstructure, and hypothesized macroscopic functional properties. Our findings inspire distinct tissue-specific structural characteristics that suggest potential structure–function relationships: The structure of labial palps mucus leads to the hypothesis that it may act as a viscous barrier-like property; mantle mucus shows features that could potentially support the formation of continuous films by a dense hydrogen-bond network; and gill mucus exhibits a porous three-dimensional network that potentially facilitates the process of respiratory and feeding. This work not only explores the material basis and potential structure–function relationships of *C. gigas* mucus as a natural biopolymer but also provides a potential theoretical framework for the design of novel marine-inspired biomimetic materials.

## 1. Introduction

Marine environments pose severe challenges to infrastructure such as offshore wind and photovoltaic power [1,2]. Corrosion is a critical industrial problem that not only causes massive economic losses—estimated at billions of dollars annually—but also generates pollutants and waste that damage aquatic and terrestrial ecosystems [3].

Simultaneously, biofouling leads to the large-scale attachment of macro-fouling organisms. Typical examples of biofouling include the heavy encrustation of ship hulls by barnacles and tubeworms, the clogging of seawater cooling pipes by mussels, and the colonization of aquaculture nets and underwater sensors by algae and hydroids [4]. These intertwined issues of corrosion and biofouling accelerate structural fatigue, increase energy consumption, and cause massive global economic and ecological losses [5,6]. Consequently, developing sustainable biomimetic materials has become a major focal point in modern materials science to mitigate these challenges.

Biomucus, a complex biomolecular gel secreted by marine invertebrates, functions fundamentally as a ‘molecular glue’ that facilitates rapid, robust, and durable underwater adhesion, while simultaneously acting as the primary biological barrier against external pathogens and physicochemical stresses [7,8]. Composed predominantly of water, proteins, polysaccharides, lipids, and inorganic ions, this secretion forms a dynamic microenvironment characterized by unique rheological properties and biological activities. For the *Crassostrea gigas*—a typical sessile marine bivalve inhabiting complex and fluctuating environments—mucus plays an indispensable role in defending against pathogen invasion, mitigating environmental stress, and promoting biomineralization [9]. Throughout the oyster’s life cycle, mucus secreted by various glands fulfills distinct and highly specialized physiological functions.

The macroscopic functionalities of marine mucus emerge from the synergistic effects of its microscopic molecular composition, network architecture, and interfacial behaviors; consequently, its functional performance is intrinsically linked to its multiscale physicochemical properties [10,11,12]. However, current research predominantly focuses on the molecular level, lacking systematic, cross-scale analyses that correlate molecular characteristics with macroscopic material properties. Notably, the majority of biochemical analyses rely on fresh or fixed mucus, largely neglecting the material properties of mucus in its desiccated state [13]. This oversight restricts a comprehensive understanding of its functional adaptability in natural environments and hinders the full exploitation of its potential as a biomaterial precursor [14]. Therefore, to bridge the gap between molecular-level investigations and macroscopic functional understanding, it is imperative to conduct further characterization of its structure, composition, and functional properties from a materials science perspective [15,16].

Consequently, this study aims to conduct a preliminary characterization of the structure, composition and functional properties of Pacific oyster mucus from a materials science perspective, with an exploratory view to bridging the gap between molecular research and an understanding of macroscopic functions [17,18]. The central objective of this research is to explore how the multiscale physicochemical properties of oyster mucus influence its macroscopic defense and adhesion functions in dynamic marine environments. Historically, academic investigations into the adhesion mechanisms of marine sessile organisms have predominantly focused on the formation of rigid inorganic mineralized cements during their larval stages, while relatively neglecting the flexible mucus defense and adhesion systems relied upon by adult organisms in complex, dynamic fluid environments. Recognizing this critical theoretical gap, this study shifts the research focus from “rigid mineralization” to “dynamic mucus interfaces” by isolating and comparing the tissue-specific mucus from three core interfaces of adult *Crassostrea gigas*: the labial palps, mantle, and gills. Deconstructing the biological antifouling effect from this physiological perspective provides a potential biomimetic paradigm for the development of traditional toxic antifouling coatings. This research not only explores the material basis of mucus as a natural biopolymer but also holds potential value and practical engineering significance for the development of possible biomimetic materials based on marine resources.

## 2. Materials and Methods

### 2.1. Ethical Statement

Pacific oysters (*C. gigas*) are marine biofouling invertebrate organisms. Ethical approval was not required for experiments with *C. gigas*.

### 2.2. Collection of Mucus Samples from C. gigas

For this experiment, adult *C. gigas* with a shell length of 15 ± 1 cm, naturally growing in the waters off Qingdao, Shandong, were collected between October and December 2024. During the first week of each month, 20 healthy individuals of consistent developmental maturity were selected (n = 60 in total). It is important to clarify that the collections over three months were not intended to establish three independent biological replicates. Because the extraction of mucus from one specific target tissue required the physical disruption and excision of the other non-target tissues to prevent cross-contamination, the mucus yield per individual was extremely limited. Therefore, to obtain a sufficient total volume for subsequent material characterizations, the mucus extracted from all 60 individuals was pooled into a single composite sample for each tissue type. This pooling strategy also served to average out individual biological variations, providing a representative baseline profile for the population.

After rinsing with sterile seawater, the oysters were acclimatized in laboratory-filtered, sterilized seawater under the following conditions: water temperature 14–16 °C and salinity 28–32‰. Prior to mucus extraction, the adductor muscle of the oysters was severed using a sterile scalpel. The mantle cavity was rinsed with phosphate-buffered saline (PBS) to remove residual seawater and impurities. Subsequently, mucus was extracted separately from the mantle, gill and labial palps. To prevent cross-contamination from the mixing of mucus from different tissues, the non-target tissues were physically disrupted and carefully excised using sterile surgical scissors and forceps prior to the extraction of mucus from the specific target tissue. The extracted mucus was centrifuged at 1000× *g* for 5 min at 4 °C to remove cellular debris and tissue fragments [19,20].

From the resulting supernatant, a total volume of 20 mL was collected for each gland type and aliquoted for specific characterizations: 5 mL was designated for scanning electron microscopy (SEM), 5 mL for Fourier-transform infrared spectroscopy (FTIR), and the remaining 10 mL was reserved for amino acid analysis. All samples were stored at −80 °C for subsequent experiments. Because all biological samples were pooled into a single composite batch per tissue type to overcome extraction limitations, no biological replicates were maintained in this study. Consequently, the characterization results represent the average composition of the pooled population.

During the extraction process, it was qualitatively observed that the crude mucus of the *C. gigas* exhibited macroscopic viscous and stringing behaviors; it could be drawn into continuous, uniform filaments using a pipette tip, suggesting potential cohesion and extensibility.

**Figure 1:**
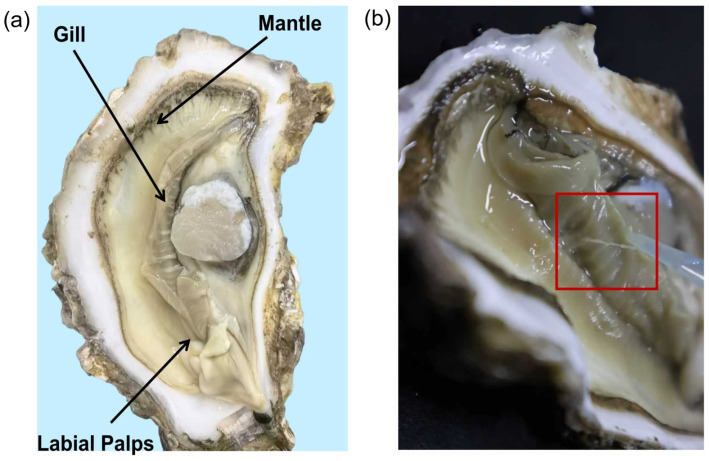
Schematic diagram of *Crassostrea gigas*: (**a**) *Crassostrea gigas*’s main anatomical organs location: Gill, Mantle, Labial Palps. (**b**) Schematic diagram of mucus filament formation. The content in the red box shows a pulled mucus strand.

Figure 1a shows a schematic diagram of the location of the *C. gigas* and its three types of glands: the gill, mantle and labial palps. Figure 1b shows a schematic diagram of mucus filament formation.

### 2.3. Freeze-Drying of Mucus Samples and Preparation of Powdera

**Figure 2:**
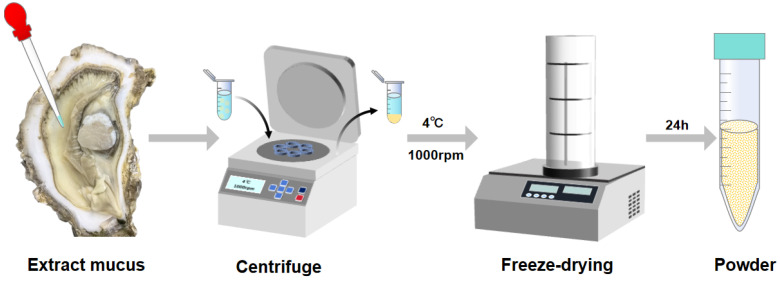
Schematic diagram of freeze-drying of *Crassostrea gigas* mucus.

Freshly collected mucus samples (10 mL per gland) were transferred into 5 mL cryovials and flash-frozen by immersion in liquid nitrogen for 10 min. To facilitate moisture sublimation while preventing cross-contamination, the vials were covered with micro-perforated aluminum foil. The samples were then placed in the chamber of a freeze-dryer pre-cooled to −80 °C. Lyophilization was conducted continuously for 24 h under a vacuum pressure of <10 Pa until a constant weight was achieved. The resulting dried mucus powder was stored in a desiccator at room temperature prior to characterization [21,22]. For subsequent analysis, the lyophilized powder from each batch was divided into two equal portions: one portion was allocated for surface morphology observation via scanning electron microscopy (SEM), and the other was reserved for chemical composition analysis using Fourier-transform infrared (FTIR) spectroscopy. This entire preparation process was performed in triplicate (n = 3) to ensure method reproducibility. Figure 2 shows the schematic diagram of freeze-drying of *Crassostrea gigas* mucus.

### 2.4. Free Amino Acid Analysis

Ten milliliters (10 mL) of each mucus sample were collected from each gland. The samples were centrifuged at 8000× *g* for 10 min at 4 °C to remove insoluble impurities. Subsequently, the supernatant was filtered through a 0.22 µm water-compatible polyethersulfone (PES) filter membrane to obtain a clear sample solution. The free amino acids in the samples were analyzed using a fully automated amino acid analyzer. This system employs ion-exchange chromatography for separation, followed by post-column ninhydrin derivatization, with detection at wavelengths of 570 nm and 440 nm (for proline) [23,24]. All experiments were performed in triplicate.

### 2.5. Scanning Electron Microscope (SEM)

The surface microstructure of the freeze-dried samples was characterized using a scanning electron microscope (SEM) to observe the morphological features and micro-architecture of the biopolymer. A small amount of the dried mucus powder was mounted onto a conductive adhesive tape; the sample was then sputter-coated with a gold layer (10 mA, 45 s) to enhance conductivity. Images were captured at an acceleration voltage of 3 kV using a secondary electron detector in high-vacuum mode [25,26].

### 2.6. Fourier-Transform Infrared Spectroscopy (FTIR)

FTIR spectra were acquired by mixing a small amount of freeze-dried powder with anhydrous potassium bromide (KBr) in a mass ratio of 1:100. The mixture was ground to a fine powder and pressed into a translucent pellet under 10 MPa pressure. Spectra were collected over a range of 400–4000 cm^−1^ with a resolution of 4 cm^−1^ and 32 scans. A pure KBr pellet was scanned for background subtraction prior to sample measurement. Baseline correction and smoothing were performed using OMNIC software (version 9.0, Thermo Fisher Scientific, Waltham, MA, USA) to facilitate functional group analysis [27,28].

### 2.7. Instrumentation

The specific instruments utilized in this study included: a scanning electron microscope (SEM; TESCAN MIRA LMS, Brno-Kohoutovice, Czech Republic) for morphological observation; a fully automated amino acid analyzer (MembraPure A300, Hennigsdorf, Germany) for amino acid quantification; and a Fourier-transform infrared spectrometer (Thermo Fisher Scientific Nicolet iS20, Waltham, MA, USA) for the identification of chemical functional groups.

## 3. Results and Discussion

### 3.1. Results of Amino Acid Analysis

**Figure 3:**
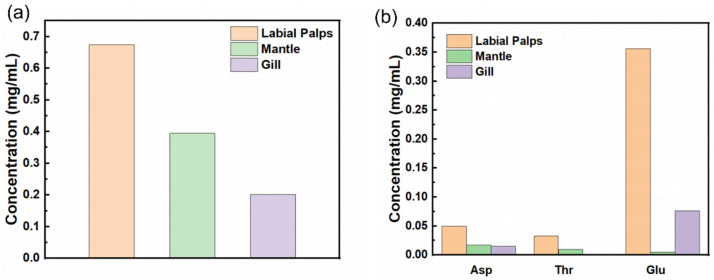
Bar chart showing the amino acid content in C. gigas mucus: (**a**) total amino acid content; (**b**) aspartic acid, threonine and glutamic acid content.

A quantitative analysis of the amino acid composition in three mucus samples—from the labial palps, mantle and gill—of the *C. gigas* revealed statistically significant differences between the samples in terms of total free amino acid content and their respective profiles (Figure 3a). Among these, the labial palps mucus exhibited the highest total free amino acid content, reaching 0.674 mg/mL, which was significantly higher than that of the mantle and gill mucus.

Regarding the specific amino acid composition, labial palps mucus exhibits an extremely high glutamic acid content, accounting for 52.8% of the total free amino acid pool and representing the dominant component in the labial mucus. In contrast, the free amino acid distribution in the mantle mucus is relatively balanced, with proline and serine being the predominant components. Meanwhile, gill mucus not only has the lowest total free amino acid content but also exhibits extremely low levels of several essential amino acids, which might suggest a comparatively lower protein concentration or metabolic turnover rate of these amino acids in this specific matrix.

While the present study focuses on the free amino acid pool rather than total protein or mucin characterization, these free metabolites constitute a crucial part of the mucosal microenvironment. We hypothesize that the variations in free amino acid profiles may reflect differences in local metabolic activity and contribute to the physicochemical properties of the mucus fluid phase:

Firstly, the total free amino acid concentration in labial palps mucus is approximately 1.7 times that of the mantle mucus and 3.3 times that of the gill mucus. If this free amino acid pool correlates with the local metabolic activity, it might imply a greater biochemical dynamism in the labial palps’ mucus. Furthermore, high concentrations of free amino acids may act as significant organic osmolytes, potentially influencing the hydration and hypothesized rheological environment of the mucus.

Furthermore, the mucus from the labial palps contains a very high proportion of acidic amino acids, with the total concentration of glutamic acid and aspartic acid reaching 0.406 mg/mL. While these are free amino acids and do not directly represent the polyelectrolyte architecture of the mucin glycoproteins, an abundance of acidic residues in the local microenvironment possibly contributes to a highly negatively charged metabolite pool. This local ionic environment may indirectly influence the swelling and hydration behavior of the surrounding mucus matrix by altering the electrostatic balance.

At the same time, labial palps mucus contains higher levels of free amino acids with strong hydration capabilities, such as threonine and aspartic acid, compared to mantle mucus and gill mucus. As free molecules, they can bind water molecules via hydrogen bonds, thereby potentially enhancing the local hydration capacity of the fluid phase. In contrast, the mantle mucus contains up to 0.08 mg/mL of free amino acids that are typically abundant in mucins, such as proline and serine; within mucin structures, the side-chain hydroxyl groups of these two amino acids may serve as primary attachment sites for O-linked sugar chains. We hypothesize that the abundance of free proline and serine might reflect a high turnover of mucin precursors in the mantle. However, it must be noted that these interpretations are based solely on free amino acid profiles. Further investigations involving total protein analysis, mucin characterization, and direct glycan or carbohydrate compositional analyses are required to further establish the macromolecular architecture, glycosylation patterns, and crosslinking behaviors of these mucus matrices.

### 3.2. Microscopic Morphological Analysis

To investigate the microscopic morphological characteristics of the mucus analyzed from the labial palps, mantle and gill of the *C. gigas*, this study utilized scanning electron microscopy (SEM) to examine freeze-dried powder samples of the three mucus types at a magnification of 10,000×. The results revealed significant differences among the three mucus powders in terms of particle morphology, spatial network structure and degree of compactness, which may reflect the heterogeneity of mucus macromolecules during the lyophilisation-induced self-assembly process. It is important to note that these morphologies represent the dehydrated state and may not fully replicate the highly hydrated structure of native mucus in vivo; however, the aggregation behavior of these freeze-dried macromolecules can still provide possible valuable insights into their intrinsic structural tendencies.

**Figure 4:**
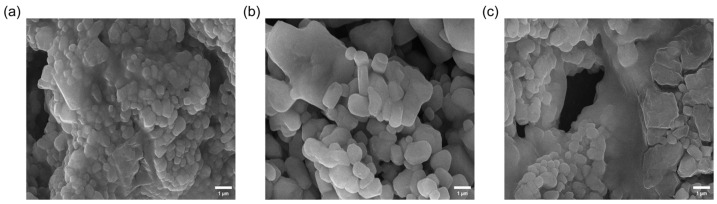
SEM images of different mucus powders from *Crassostrea gigas*: (**a**) labial palps; (**b**) mantle; (**c**) gill.

As shown in Figure 4a, the overall spatial structure of the labial palps mucus powder is highly compact, with comparatively low porosity. Morphologically, it consists of closely packed, spherical or polyhedral particles, with few lamellar structures. The particle surfaces are relatively rough, and small particles can be observed locally adhering to larger particles, forming a hierarchical composite structure. These morphological characteristics potentially indicate a complex aggregation of proteins or polysaccharides, whilst crystals or regular lamellar structures are relatively scarce. This dense particle packing, combined with the rough, multi-level surface structure, collectively forms a solid network in the dried state. Functionally, we speculatively hypothesize that such a dense macromolecular aggregation tendency observed in the lyophilized state could potentially facilitate the formation of what might act as an effective barrier-like property at the tissue–environment interface when hydrated, which may be related to specific physiological requirements, though further permeability studies on native mucus are needed to suggest this indirect inference.

As shown in Figure 4b, the mantle mucus powder exhibits a microstructure that is markedly different from that of the labial palps and gill mucus powders; its structure is the most uniform and dense of the three powders, with relatively smooth particle surfaces and rounded edges. The particles are closely packed and even partially fused in places, with virtually no discernible pores, and the powder shows no obvious crystalline structure. These characteristics suggest that macromolecular complexes, such as mucins, within the mantle mucus may possess a more regular self-assembled arrangement, upon freeze-drying, with potentially ordered stacking of their polysaccharide molecular chains being more ordered. This smooth, dense and continuous structure in the lyophilized state possibly suggests a highly cohesive macromolecular matrix. Consequently, it is speculatively hypothesized that mantle mucus may be adapted to potentially be associated with forming barrier-like properties against environmental stressors, reflecting its dense molecular packing observed post-dehydration. However, this morphology–function link remains an indirect deduction.

As shown in Figure 4c, the gill mucus powder exhibits a classic porous three-dimensional network structure, with highly heterogeneous microstructural components consisting primarily of a mixture of rough-surfaced, spherical and cluster-like particles, as well as some well-defined lamellar structures. This structure, characterized by abundant pores and a mixture of diverse morphologies, possibly increases the specific surface area of the gill mucus in its solid state. Consequently, the loose network structure is speculatively hypothesized to provide the gill mucus with potential for enhanced adsorption and encapsulation capabilities upon hydration. This loose three-dimensional network may suggest a less densely cross-linked macromolecular arrangement compared to the other two tissues. While this specific drying behavior might indirectly reflect a native composition potentially adapted for high permeability and rapid mass transfer rather than acting as a dense barrier-like property, direct observations and measurements of hydrated native mucus are essential to substantiate this speculative hypothesis and avoid over-interpreting the powder morphology.

### 3.3. Fourier-Transform Infrared Spectral Characterization

**Figure 5:**
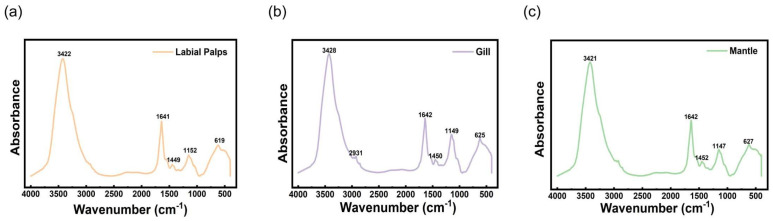
FTIR images of different mucus powders from *Crassostrea gigas*: (**a**) labial palps; (**b**) gill; (**c**) mantle.

As shown in Figure 5a, labial palps mucus exhibited the highest absorbance response among the three mucus samples, potentially suggesting a relatively high organic matter content. The absorption peak intensity of the labial palps mucus at 3422.85 cm^−1^ reached 0.258, and the Amide I band at 1640.33 cm^−1^ exhibited the sharpest peak shape and highest intensity, indicating the presence of high concentrations of protein components relative to the other samples. Furthermore, the characteristic polysaccharide peak of the labial palps mucus at 1151.62 cm^−1^ exhibits a marked blue shift; such a shift is typically associated with changes in the microenvironment of the polysaccharide chains or differences in the configuration of glycosidic bonds. As the core organ of filter-feeding, the high protein content and strong intermolecular hydrogen bonding interactions of the labial palps’ mucus possibly suggest a highly cohesive molecular architecture in the lyophilized state.

As shown in Figure 5b, the spectrum of gill mucus exhibits typical glycoprotein characteristics. The most prominent feature is a broad and intense absorption peak at 3424.99 cm^−1^, which arises from the superposition of the N–H stretching vibration in the protein amide A band and the hydroxyl stretching vibration in the polysaccharides. Compared with the spectra of mucus from the labial palps and mantle, the peak positions in the gill mucus spectrum are slightly shifted towards higher wavenumbers, indicating a relatively weaker degree of internal hydrogen bonding within the lyophilized sample. In the fingerprint region, the intensity of the amide I band at 1640.39 cm^−1^ is moderate, suggesting the presence of a stable protein backbone in the gill mucus. Furthermore, the ratio of the peak intensity at 1450.04 cm^−1^ to that of the polysaccharide C–O–C stretching vibration peak at 1148.04 cm^−1^ is similar, reflecting a relatively balanced distribution of protein side chains and mucopolysaccharide components in the gill mucus.

As shown in Figure 5c, the spectral profile of the mantle mucus lies between that of the labial palps mucus and the gill mucus, sharing some common features whilst exhibiting its own unique characteristics. The peak of the broad absorption band near 3421.95 cm^−1^ is shifted towards lower wavenumbers compared to the labial palps and gill mucus, indicating that intermolecular hydrogen bonding interactions are most pronounced in the mantle mucus in its solid state. The intensity of the amide I band at 1640.24 cm^−1^ is higher than that of gill mucus but lower than that of labial palps mucus, suggesting that the content of secondary protein structures in the mantle mucus is intermediate. Furthermore, a distinctive feature of the mantle mucus spectrum is that the peak at 1450.15 cm^−1^ is sharper than that of gill mucus, and a clear characteristic peak appears near 1149.99 cm^−1^; this region possibly corresponds to the stretching vibrations of C-O-C or C-O bonds in polysaccharide compounds, indicating that mantle mucus may contain a significant polysaccharide component. The infrared spectrum of the mantle mucus reveals a complex chemical composition that combines certain protein characteristics with significant polysaccharide features, mediated by strong intermolecular hydrogen bonding interactions. This close molecular association and specific compositional structure may possibly confer enhanced structural stability at the molecular level.

In summary, the FTIR spectra of the three glandular mucus samples show a high degree of consistency in their overall profiles, all consisting of proteins and glycosaminoglycans, suggesting the conservation of the basic chemical composition of mucus from different glands in the *C. gigas*. However, the significant differences in absorbance intensity reflect variations in the relative abundance of these components among the different glands, with the labial palps exhibiting the highest absorbance response and the highest accumulation of organic matter. A comparison of the peak intensities at 1640 cm^−1^ and 1150 cm^−1^ reveals that the labial palps mucus may exhibit the highest accumulation of proteins and polysaccharides. Minor shifts in peak positions in the 3420 cm^−1^ region reflect differences in the hydrogen-bond networks within the solid state. Notably, the absorption peak of the mantle mucus shifted towards the lowest wavenumber, possibly suggesting the intermolecular hydrogen-bond interactions and a highly cohesive intermolecular network within this dried system. While direct mechanical and rheological measurements of hydrated mucus are necessary to suggest its physical performance, this molecular structural characteristic observed here is theoretically consistent with its hypothesized physiological role. The barrier-like properties may potentially aid in resisting mechanical and biological disturbances from the environment. Nevertheless, extrapolating macroscopic mechanical or biological defense properties directly from these spectral features should be strictly avoided.

### 3.4. Limitations of the Study

While providing foundational insights, this study acknowledges two key limitations. Firstly, due to limited extraction volumes, samples were pooled per tissue type. This lack of biological replicates means the results reflect average profiles, precluding the assessment of individual variability. Secondly, this work is framed as a preliminary descriptive characterization rather than a full functional analysis. Consequently, any hypothesized macroscopic functions remain highly speculative, as they are indirectly inferred from the microstructural features of dehydrated, freeze-dried samples. Future studies incorporating independent biological replicates and direct functional assays on native hydrated mucus are potentially essential to validate these structure–function relationships.

## 4. Conclusions

In summary, this study preliminarily characterized the biochemical composition and microstructural properties of the pooled three types of mucus secreted by the labial palps, mantle and gill mucus of the *C. gigas*, as well as the basic structural features of their freeze-dried powders. The main conclusions based on the bulk characterization are as follows:

The labial palps mucus is characterized by a very high proportion of glutamic acid and acidic amino acids, alongside the highest organic matter concentration and protein abundance among the three pooled samples. This compositional advantage suggests the potential for polyelectrolyte effects and strong hydration, which may possibly contribute to the hypothesized formation of a highly cohesive molecular network that could potentially act as a barrier-like property.

The mantle mucus is dominated by proline and serine, exhibiting distinct polysaccharide characteristics and the densest hydrogen-bond network among the three types. It possesses moderate viscosity and displays a uniform, smooth, and pore-free microstructure, which may lead to the hypothesis that it could potentially be suitable for forming continuous films with hypothesized lubricating and protective functions.

The gill mucus exhibits the lowest total amino acid content and protein concentration; its protein and polysaccharide distribution is balanced, and its hydrogen bonding is relatively loose. Notably, its loose, porous three-dimensional network—composed of heterogeneous particles and lamellar structures—implies a potentially high specific surface area.

In conclusion, this study provides a preliminary descriptive characterization of *C. gigas* mucus. Given the use of pooled samples, the current findings represent average population profiles; therefore, future research incorporating independent biological replicates and direct functional assays is essential to validate these hypothesized structure–function relationships.

## Data Availability

The original contributions presented in this study are included in the article. Further inquiries can be directed to the corresponding author.
